# *MC4R* variants rs12970134 and rs17782313 are associated with obese polycystic ovary syndrome patients in the Western region of Saudi Arabia

**DOI:** 10.1186/s12881-019-0876-x

**Published:** 2019-08-20

**Authors:** Asma A. Batarfi, Najlaa Filimban, Osama S. Bajouh, Ashraf Dallol, Adeel G. Chaudhary, Sherin Bakhashab

**Affiliations:** 1 Biochemistry Department, King Abdulaziz University, P.O. Box 80218, Abdullah Sulayman St., Jeddah, 21589 Saudi Arabia; 2 Centre of Innovation in Personalized Medicine, King Abdulaziz University, P.O. Box 80216, Jeddah, Saudi Arabia; 3 Department of Obstetrics and Gynaecology, Faculty of Medicine, King Abdulaziz University, P.O. Box 80205, Jeddah, Saudi Arabia

**Keywords:** BMI, Polycystic ovary syndrome, *MC4R*, SNP

## Abstract

**Background:**

Polycystic ovary syndrome (PCOS) is a common endocrine disorder causing infertility in reproductive-age women. The cause of PCOS is not fully understood but it is thought to be influenced by environmental and genetic factors. Obesity is greatly related to PCOS and its reduction is one of the major aims in treating PCOS. Melanocortin 4 receptor (*MC4R*) gene polymorphisms were detected to be associated with different levels of obesity. Therefore, we aimed to determine the genotype and allele frequency of *MC4R* variants rs12970134 (A/G) and rs17782313 (C/T) in PCOS and investigate their association with PCOS and its clinical variables.

**Methods:**

A case-control study was conducted on 189 women, consisting of 95 PCOS cases and 94 controls. Genotyping was performed by real-time polymerase chain reaction (PCR) using TaqMan™ Genotyping assays. Quantitative data were presented as (median ± interquartile range (IQR) whereas qualitative data were presented as frequencies. The chi-squared test was used to observe the difference between SNPs within the study groups (PCOS and control subjects). Multinomial logistic regression was used to test the risk of obesity and development of PCOS considering *p* < 0.05 is statistically significant.

**Results:**

Rs12970134 and rs17782313 are significantly associated with body mass index (BMI, kg/m^2^, *p* < 0.0001) in PCOS women but not associated with PCOS itself. Risk alleles in our population are A in rs12970134 and C in rs17782313 that are associated with high BMI (> 30 kg/m^2^) in obese women with PCOS (OR = 1.348, *p* = 0.002 and OR = 1.364, *p* = 0.002 respectively) in the homozygous state. In addition, we found that the other genotypes for non-obese PCOS group, AG/GG for rs12970134 and CT/TT for rs17782313, are associated with hirsutism, loss of hair, hyperandrogenism and anti-Müllerian hormone in PCOS.

**Conclusions:**

These findings demonstrate that *MC4R* single nucleotide polymorphisms, rs12970134 and rs17782313, are correlated with elevated BMI in PCOS but are not causative factors for PCOS among women in the western region of Saudi Arabia. Moreover, the reverse genotypes are associated with major clinical variants in non-obese (< 30 kg/m^2^) PCOS patients may demonstrate a poor prognosis for this group.

## Background

Polycystic ovary syndrome (PCOS) is a common heterogeneous endocrine disorder with prevalence up to 15–20% in reproductive-age women worldwide depending on which criteria are used [1]. The aetiology of PCOS is still unclear, but it is thought to be associated with environmental factors such as lifestyle and genetic factors [2, 3]. There are three major criteria for PCOS diagnosis: one criterion raised by the National Institutes of Health (NIH) which includes only the existence of hyperandrogenism (HA) and oligo/anovulation (OA), the second criterion was introduced by Androgen Excess Society (AES) which defined PCOS as HA with ovarian dysfunction or polycystic ovaries, and the Rotterdam criterion, which is widely used for PCOS diagnosis [4–6]. However, in the Rotterdam criterion, PCOS is diagnosed by the appearance of two out of three clinical features: OA, clinical or biochemical HA and polycystic ovarian morphology (PCOM) [7]. PCOM is characterized by the presence of 12 or more follicles in either ovary measuring 2–9 mm in diameter [8]. Furthermore, PCOS patients have an abnormal luteinizing hormone (LH) to follicle stimulating hormone (FSH) ratio [7]. Serum anti-Müllerian hormone (AMH) also has been used as a marker for the ovarian antral follicle number and thus offers a diagnostic tool for PCOS, especially when accurate ultrasonic data are not available [9]. PCOS is related to endocrine-metabolic disorders, thus women with this syndrome are at high risk of dyslipidaemia, cardiovascular disease, insulin resistance, glucose intolerance, type 2 diabetes mellitus [10], and obesity [11]. Obesity is associated with PCOS, but its causal role has not yet been determined [11], although other explanations involve opposite causality, that is, PCOS raises susceptibility to weight gain [12]. The prevalence of obesity among PCOS patients in Saudi Arabia is 51% as reported previously by Al-Ruhaily et al. [13]. Moreover, 50 to 80% of women with PCOS in other countries are obese [14, 15]. Obesity increases the severity of PCOS and may play a central role in the development of HA and chronic anovulation [16]. There is an association of insulin resistance with PCOS but the molecular mechanisms of insulin resistance in PCOS vary from other insulin-resistance disorders such as obesity and diabetes [11, 17]. PCOS is a common, complex genetic disorder with multifactorial aetiology, in which a variety of genes interact with environmental factors to cause the disease [18]. The hereditary basis of PCOS increases the risk of the prevalence of PCOS among family women [10]. Recent genome-wide association studies (GWAS) have identified the susceptibility loci for many complex genetic diseases, diabetes and obesity [19–23]. These GWAS studies have detected variations in or near different genes, among them fat mass- and obesity-associated gene alpha-ketoglutarate dependent dioxygenase and melanocortin 4 receptor (*MC4R*) in obesity [19, 21–23]. *MC4R* is located on chromosome *18q22*, and it is the most common genetic cause for inherited morbid obesity [10, 24]. In addition, it is an important regulator in central melanocortin neuronal pathways [25]. Previous studies demonstrated several *MC4R* variants and common genetic polymorphisms near the *MC4R* gene contributing to common obesity [22, 26, 27]. Among these variants, rs12970134 (A/G) and rs17782313 (C/T) have been studied most often. The majority of studies showed a significant association of these variants with obesity-related traits, whereas some studies revealed non-significant association [28–31]. Moreover, the association of rs12970134 *MC4R* variant with insulin resistance has been documented [26].

To our knowledge, no previous study has been conducted on the effect of *MC4R* polymorphism on PCOS among the Saudi population. Thus, in this study we aimed to determine the association between *MC4R* gene variants (rs12970134 and rs17782313) because these are risk factors in obesity; and different clinical characteristics of PCOS. This will aid in understanding the susceptibility of genetic variations in PCOS and their correlation to obesity. Therefore, we will be able to identify specific risk variants for PCOS and a better understanding of the genetic preposition for this disorder.

## Methods

### Study design

This is a case-control, observational and randomised study that was conducted from the Obstetrics and Gynaecology Clinics, King Abdulaziz University Hospital and the Centre of Innovation in Personalized Medicine (CIPM), KAU, Jeddah, Saudi Arabia, clinical database from 2016 up to 2018. The total population was 400 cases after applying the exclusion criteria. The exclusion criteria were as follows:

1. A condition with reproductive symptoms similar to PCOS, including congenital adrenal hyperplasia, Cushing syndrome, hyperprolactinemia, thyroid disease, and androgen-secreting tumours.
2. Chronic diseases, including diabetes, cardiovascular disease.
3. Any other female infertility factor.

The sample size was calculated using raosoft (www.raosoft.com) which indicated 196 women of reproductive age between 18 and 38 years old. The study was approved by the Biomedical Ethics Unit, Faculty of Medicine, KAU (approval number: 407–15) and written informed consent was obtained from participants prior to sample collection. The study was conducted in accordance with the Declaration of Helsinki.

The case group was 98 PCOS selected patients, diagnosed according to the Rotterdam Criteria Consensus and the control group was 98 women with normal ovulation. In days 2–4 of the menstrual cycle transvaginal or abdominal ultrasound was performed using a SonixTouch machine (Ultrasonix Medical Corporation, Richmond, BC, Canada) and serum hormonal levels for LH and FSH were measured with an automated multi-analysis system using electrochemiluminescence immunoassay (ECLIA) kits (Roche, Basel, Switzerland). Serum AMH levels were determined using an enzyme-linked immunosorbent assay using Ultra-Sensitive Anti-Müllerian hormone/Müllerian inhibiting substance (US AMH/MIS) kit (AnshLabs, Webster, TX, USA) according to the manufacturer’s instructions. PCOM was confirmed by the presence of at least 12 follicles measuring between 2 and 9 mm in diameter in one ovary. Body mass index (BMI) was determined by a person’s weight in kilograms over square of height in m^2^. HA was defined by the presence of hirsutism, acne or androgenic alopecia [32, 33]. Samples with poor quality were excluded from the statistical analysis to achieve 95 cases and 94 controls.

### Genotyping

Genomic DNA was isolated from peripheral whole blood using the QIAamp DNA Mini Blood Kit (Qiagen, Hilden, Germany) according to the manufacturer’s instructions. Genotyping of the *MC4R* variants rs12970134 (assay ID: C_3058722_10) and rs17782313 (assay ID: C_32667060_10) was performed using TaqMan™ single nucleotide polymorphism (SNP) Genotyping Assays (Thermo Fisher Scientific, Waltham, MA, USA). Allelic PCR products were analysed using QuantStudio 12 K Flex Real-Time PCR System (Thermo Fisher Scientific).

### Statistical analysis

A normality test was performed to show that the data do not follow the normal distribution. Quantitative data are presented as (median ± interquartile range (IQR)), *p*-values were calculated using the Mann–Whitney test for non-normal distribution data, whereas qualitative data are presented as frequencies. Genotypes and allelic frequencies were calculated within our cohort population to determine the significant differences between PCOS and control groups. The chi-squared test was used to observe the difference between SNPs within the study groups (PCOS and control subjects). Differences between SNPs and continuous variables were tested using the Kruskal–Wallis test, the *p*-value was adjusted using the Bonferroni correction between pairwise comparisons. Further, multinomial logistic regression was used to determine the risk of high BMI and development of PCOS. The correlation of *MC4R* variants with PCOS symptoms was calculated using binary logistic regression for patients with regard to the control. *p* < 0.05 was considered statistically significant. Data analysis was conducted using the IBM SPSS software version 24 (SPSS™ Inc., Armonk, NY, USA).

## Results

The clinical characteristics of the participants are summarized in Table 1.

**Table 1:** Clinical characteristics of PCOS patients and control subjects

Variable	Control (*n* = 94)	PCOS patients (*n* = 95)	*p*-value
Age (years)	21.0 ± 3	22.0 ± 9.0	0.015*
BMI (kg/m^2^)	22.7 ± 5.9	24.56 ± 7.34	0.003**
LH (IU/ml)	5.7 ± 5.7	9.0 ± 8.7	0.001**
FSH (IU/ml)	4.6 ± 2.6	4.8 ± 2.4	0.339
LH/FSH ratio	1.2 ± 1.5	1.9 ± 1.6	0.001**
AMH (ng/ml)	2.3 ± 1.4	4.8 ± 4.76	< 0.0001***

Values are expressed as median ± IQR, *p*-values were calculated using the Mann–Whitney test for non-normal distribution data. *p*-value < 0.05 is statistically significant. *BMI* body mass index, *LH* luteinizing hormone, *FSH* follicle stimulating hormone, *AMH* anti-Müllerian hormone. *p < 0.05, **p < 0.01, ***p < 0.001

### Allele and genotype frequency

The distribution of rs12970134 and rs17782313 genotype and allele frequencies is listed in Table 2. *MC4R* rs12970134 genotypes (AA, AG and GG) frequencies were estimated for the PCOS group as 6.3, 40.0 and 53.7%, respectively, whereas for the control group as 5.3, 37.2 and 57.4%, respectively. Allele A and G frequencies for the same variant were estimated as 26.3 and 73.7%, respectively for the PCOS group and 23.9 and 76.1%, respectively, for the control group. However, *MC4R* rs17782313 genotypes (CC, CT and TT) frequencies were calculated for the PCOS group as 7.4, 40.1 and 51.6%, respectively, whereas for the control group as 4.3, 36.2 and 59.6%, respectively. Moreover, allele C and T frequencies were estimated as 27.9 and 72.1%, respectively, for the PCOS group and 22.3 and 77.7%, respectively, for the control group.

**Table 2:** Genotype and allele frequencies for *MC4R* variants rs12970134 and rs17782313

SNP	rs12970134			*p*-value	rs17782313			*p*-value
Genotype frequency	AA	AG	GG		CC	CT	TT	
Patient (*n* = 95)	6 (6.3%)	38 (40.0%)	51 (53.7%)	**0.86**	7 (7.4%)	39 (40.1%)	49 (51.6%)	**0.44**
Control (*n* = 94)	5 (5.3%)	35 (37.2%)	54 (57.4%)		4 (4.3%)	34 (36.2%)	56 (59.6%)	
Total = 189	11 (5.8%)	73 (55.6%)	105 (38.6%)		11 (5.8%)	73 (55.6%)	105 (38.6%)	
Allele frequency	A	G			C	T		
PCOS (*n* = 95)	50 (26.3%)	140 (73.7%)			53 (27.9%)	137 (72.1%)		
Control (*n* = 94)	45 (23.9%)	143 (76.1%)			42 (22.3%)	146 (77.7%)		

*p*-value was calculated using the chi-squared test

### The association of MC4R variants rs12970134 and rs17782313 with PCOS

There is no direct association between the *MC4R* SNPs and PCOS, in addition to the other clinical variables such as acne, hirsutism, loss of hair, HA**,** PCOM and OA by using the chi-squared test. There was significant difference between BMI (> 30 kg/m^2^) and *MC4R* variants rs12970134 and rs17782313 only in the patients’ group (*p* = 0.003 and *p* = 0.001, respectively) as shown in Table 3. In rs12970134, significant variance was detected by comparing A/A to A/G and A/A to G/G, whereas in rs17782313 significant differences were observed between C/C compared with C/T and C/C compared to T/T.

**Table 3:** Association between *MC4R* variants rs12970134 and rs17782313 and the multiple variables

rs12970134								
Variables	Patient (*n* = 95)			*p*-value	Control (*n* = 94)			*p*-value
	A/A	A/G	G/G		A/A	A/G	G/G	
BMI (kg/m^2^)	33.5 ± 9.4	24.5 ± 6.8	23.5 ± 6.6	0.003**	23.2 ± 13.8	21.1 ± 5.5	23.1 ± 7.3	0.941
AMH (ng/ml)	5.6 ± 5.6	4.9 ± 5.5	4.7 ± 4.4	0.953	2.2 ± 0.0	2.3 ± 1.9	2.3 ± 1.4	0.482
LH (IU/ml)	9.4 ± 3.4	8.8 ± 8.9	8.3 ± 9.6	0.941	3.4 ± 5.7	6.6 ± 6.2	5.6 ± 5.3	0.224
FSH (IU/ml)	4.6 ± 2.7	5.3 ± 2.9	4.7 ± 2.2	0.300	3.3 ± 4.3	4.6 ± 2.5	4.9 ± 2.6	0.502
LH/FSH ratio	2.1 ± 1.3	2.0 ± 1.6	1.7 ± 1.7	0.926	0.8 ± 1.1	1.7 ± 1.4	1.2 ± 1.3	0.325
rs17782313								
Variables	Patient (*n* = 95)			*p*-value	Control (*n* = 94)			*p*-value
	C/C	C/T	T/T		C/C	C/T	T/T	
BMI (kg/m^2^)	34.9 ± 6.0	24.1 ± 5.6	23.5 ± 7.1	0.001***	23.6 ± 17.3	21.4 ± 7.0	23 ± 5.8	0.794
AMH (ng/ml)	4.3 ± 5.0	4.9 ± 5.5	4.7 ± 4.5	0.982	2.3 ± 0.0	2.3 ± 1.9	2.3 ± 1.6	0.881
LH (IU/ml)	9.3 ± 6.1	8.8 ± 9.2	8.4 ± 9.6	0.975	4.4 ± 5.9	6.1 ± 6.4	5.6 ± 5.7	0.573
FSH (IU/ml)	4.6 ± 2.4	5.2 ± 2.7	4.8 ± 2.3	0.690	3.8 ± 4.8	4.5 ± 2.8	4.9 ± 2.3	0.573
LH/FSH ratio	1.8 ± 1.6	2.2 ± 1.8	1.7 ± 1.6	0.998	1.0 ± 1.3	1.7 ± 1.4	1.1 ± 1.4	0.367

Quantitative data are presented as median ± IQR. Kruskal–Wallis was applied and the *p*-value was adjusted by Bonferroni correction between pairwise comparisons. *AMH* anti-Müllerian hormone, *BMI* body mass index, *FSH* follicle stimulating hormone, *LH* luteinizing hormone. **p < 0.01, ***p < 0.001

Furthermore, patients with A/A genotype are more likely to have high BMI (OR = 1.348, *p* = 0.002) than patients with G/G genotype in the rs12970134 variant using a multinomial logistic regression test. This indicates that A could be a risk allele of high BMI in PCOS women, whilst, in rs17782313 the test indicated that patients with the C/C genotype are more likely to have higher BMI (OR = 1.364, *p* = 0.002) than patients with the T/T genotype. This means that the effect of C allele on BMI should be in the homozygous state among women with PCOS (Table 4). No significant correlation with other study variables was detected in the PCOS group.

**Table 4:** Impact of *MC4R* variants rs12970134 and rs17782313 on PCOS using a multinomial logistic regression test

SNP	Variable	Group	Genotype	B	*p*-value	OR (95% CI)
rs12970134 ^a^	BMI (kg/m^2^)	Control (*n* = 94)	A/G	−0.003	0.935	0.997 (0.921–1.078)
			A/A	0.052	0.48	1.053 (0.912–1.216)
		Patient (*n* = 95)	A/G	0.027	0.553	1.027 (0.94–1.122)
			A/A	0.298	0.002**	1.348 (1.113–1.631)
rs17782313 ^b^	BMI (kg/m^2^)	Control (*n* = 94)	C/T	0.035	0.935	1.035 (0.957–1.12)
			C/C	0.102	0.48	1.108 (0.953–1.287)
		Patient (*n* = 95)	C/T	−0.008	0.553	0.992 (0.906–1.086)
			C/C	0.311	0.002**	1.364 (1.121–1.661)

^a^The reference category is G/G

^b^The reference category is T/T

**p < 0.01

Moreover, the association of *MC4R* variants rs12970134 and rs17782313 with PCOS variables acne, loss of hair, hirsutism, PCOM, OA, BMI, HA, AMH, LH, FSH and LH/FSH ratio was studied using binary logistic regression. The analysis revealed a significant association between rs12970134 and hirsutism, loss of hair, HA and AMH but with the reverse genotype that is associated with high BMI. Patients with A/G and G/G genotypes are more likely to have loss of hair, hirsutism, HA and AMH than the control group with A/G and G/G genotypes as summarized in Table 5. Therefore, in rs12970134 variant, allele G is a risk allele that is associated with hirsutism, loss of hair, HA and high AMH level and only one allele is enough to manifest these symptoms. In addition, there is a significant association between rs17782313 and hirsutism, loss of hair, HA and AMH. Patients with C/T and T/T genotypes are more likely to have hirsutism, loss of hair, hirsutism, HA and AMH than the control group with the same genotypes (Table 5). Hence, T allele in rs17782313 is potentially a risk allele associated with hirsutism, loss of hair, HA and high AMH level in PCOS patients and only one allele is enough to produce these symptoms.

**Table 5:** The correlation of *MC4R* variants rs12970134 and rs17782313 with PCOS symptoms using binary logistic regression for patients with regard to controls

SNP	Variable	Genotype	B	*p*-value	OR (95% CI)
rs12970134	hirsutism	A/A	22.119	0.999	
		A/G	1.713	0.014*	5.547 (1.424–21.612)
		G/G	3.883	< 0.0001***	48.583 (6.185–381.647)
	loss of hair	A/A	2.773	0.08	16 (0.722–354.803)
		A/G	1.787	0.001**	5.971 (2.013–17.705)
		G/G	1.13	0.014**	3.096 (1.255–7.641)
	HA	A/A	1.792	0.214	6.000 (0.354–101.568)
		A/G	1.727	.001**	5.625 (2.004–15.792)
		G/G	1.438	.001**	4.211 (1.817–9.758)
	AMH (ng/ml)	A/A	101.39	0.993	
		A/G	0.599	0.004**	1.821 (1.215–2.730)
		G/G	0.526	< 0.0001***	1.693 (1.285–2.231)
rs17782313	hirsutism	C/C	21.896	0.999	
		C/T	2.119	0.008**	8.32 (1.717–40.311)
		T/T	3.162	< 0.0001***	23.625 (5.126–108.889)
	loss of hair	C/C	2.708	0.089	15 (0.663–339.548)
		C/T	1.646	0.003**	5.185 (1.747–15.386)
		T/T	1.212	0.009**	3.362 (1.361–8.303)
	HA	C/C	1.609	0.278	5.000 (0.273–91.518)
		C/T	1.776	.001**	5.907 (2.116–16.492)
		T/T	1.412	.001**	4.103 (1.768–9.518)
	AMH (ng/ml)	C/C	101.26	0.993	
		C/T	0.598	0.003**	1.818 (1.227–2.694)
		T/T	0.536	< 0.0001***	1.71 (1.293–2.261)

*p < 0.05, **p < 0.01, ***p < 0.001

## Discussion

In this study, we examined *MC4R* variants’ (rs12970134 and rs17782313) contribution to obesity and their influence on the susceptibility to PCOS. We detected a highly significant association between both SNPs and high BMI in PCOS patients in Saudi Arabia. However, there was no direct association between those *MC4R* SNPs and PCOS. This is in concordance with previous studies among PCOS women, they have perceived that there is an association between *MC4R* SNPs (rs12970134 and rs17782313) and obesity whereas the SNPs are not risk factors for PCOS [34, 35]. Alternatively, a German study on PCOS opposed our findings because they found no association between *MC4R* SNPs (rs12970134 and rs17782313) and BMI [36], although the mean of BMI in the recruited patients > 30 kg/m^2^. This variation could be attributed to ethnic differences and environmental stimuli.

Our study also showed that A and C alleles in rs12970134 and rs17782313, respectively, are the risk alleles that are associated with high BMI in PCOS women in Saudi Arabia. These alleles were anticipated as global risk alleles for *MC4R* variants in obesity [26, 27]. Recently, the rs17782313 variant was detected to be associated with moderate obesity in the Saudi population [37]. To our knowledge, we are the first group to predict the risk alleles in these variants that are associated with PCOS symptoms. Thus, the G and T alleles in rs12970134 and rs17782313, respectively, in homozygous or heterozygous states among non-obese patients are associated with PCOS symptoms including hirsutism, loss of hair, HA and high AMH. Previously, Mahmoudi et al. studied the association between six *MC4R* variants and female pattern hair loss but in non-PCOS subjects; none of the genotyped variants displayed any significant association [38]. Lean women with PCOS have different phenotypic and clinical characteristics than obese and thus, till now, there is debate on their characteristics [39]. Therefore, the characterization of different sub-populations is of great importance to manipulate specific management and treatment.

## Conclusion

In conclusion, this genetic study reveals that *MC4R* variants (rs12970134 and rs17782313) with genotypes A/A and C/C, respectively, were highly associated with obesity in PCOS. Different genotypes of these variants were associated with clinical characteristics in non-obese PCOS women such as hirsutism, loss of hair, HA or high AMH. Therefore, high BMI (> 30 kg/m^2^) is the major cause of PCOS in Saudi Arabia. In addition, poor prognosis in the lean PCOS group could be associated with reverse genotypes.

## Data Availability

Not applicable

## Abbreviations

AES: Androgen Excess Society
AMH: Anti-Müllerian hormone
BMI: Body mass index
FSH: Follicle stimulating hormone
GWAS: Genome-wide association studies
HA: Hyperandrogenism
LH: Luteinizing hormone
MC4R: Melanocortin 4 receptor
NIH: National Institutes of Health
OA: Oligo/anovulation
OR: Odds ratio
PCOM: Polycystic ovarian morphology
PCOS: Polycystic ovary syndrome
SNP: Single nucleotide polymorphism
